# Tourism experiences reduce the risk of cognitive impairment in the Chinese older adult: a prospective cohort study

**DOI:** 10.3389/fpubh.2023.1271319

**Published:** 2023-10-24

**Authors:** Qian Li, Zheng Guo, Fangli Hu, Mengfei Xiao, Qiang Zhang, Jun Wen, Tianyu Ying, Danni Zheng, Youxin Wang, Song Yang, Haifeng Hou

**Affiliations:** ^1^School of Public Health, Shandong First Medical University and Shandong Academy of Medical Sciences, Jinan, China; ^2^Division of Epidemiology, Department of Medicine, Vanderbilt Epidemiology Center, Vanderbilt-Ingram Cancer Center, Vanderbilt University Medical Center, Nashville, TN, United States; ^3^Centre for Precision Health, Edith Cowan University, Joondalup, WA, Australia; ^4^Department of Thoracic Surgery, The Second Affiliated Hospital of Shandong First Medical University, Taian, China; ^5^School of Business and Law, Edith Cowan University, Joondalup, WA, Australia; ^6^Department of Tourism and Hotel Management, Zhejiang University, Hangzhou, China; ^7^Department of Tourism, Fudan University, Shanghai, China; ^8^Beijing Key Laboratory of Clinical Epidemiology, School of Public Health, Capital Medical University, Beijing, China; ^9^Department of Endocrinology, The Affiliated Taian City Central Hospital of Qingdao University, Taian, China

**Keywords:** tourism, cognitive impairment, dementia, incidence, prevention, cohort study

## Abstract

**Background:**

Given the etiological complexity of cognitive impairment, no effective cure currently exists for precise treatment of dementia. Although scholars have noted tourism’s potential role in managing cognitive impairment and mild dementia, more robust empirical investigation is needed in this area. This study aimed to examine the associations between tourism and cognitive impairment and dementia in older Chinese adults.

**Method:**

From a nationwide community-based cohort, 6,717 individuals aged ≥60 were recruited from 2011 to 2014, of whom 669 (9.96%) had had at least one tourism experience in the 2 years prior to enrollment. All the participants were then prospectively followed up until 2018. The association between tourism and cognitive impairment was examined by the Cox proportional hazards regression model. The adjusted hazard ratio (aHR) and its 95% confidence interval (CI) were calculated to evaluate the effect of tourism experience on cognitive impairment and dementia.

**Results:**

A total of 1,416 individuals were newly diagnosed with cognitive impairment and 139 individuals with dementia onset during follow-up. The incidence of cognitive impairment was significantly lower among participants with tourism experiences (316.94 per 10,000 person-years) than those without such experiences (552.38 per 10,000 person-years). Cox regression showed that tourism decreased the risk of cognitive impairment (aHR = 0.69, 95% CI: 0.41–0.62) when adjusted for behavioral covariates and characteristics. Compared with participants without tourism experiences, those with 1, 2, and ≥3 tourism experiences had a lower risk of cognitive impairment with the aHRs of 0.72 (95% CI: 0.52–0.99), 0.65 (0.42–1.01), and 0.68 (0.44–0.98), respectively. Tourism experiences also reduced participants’ risk of dementia (aHR = 0.41, 95% CI: 0.19–0.89).

**Conclusion:**

Our findings demonstrated associations between tourism and reduced risks of cognitive impairment and dementia in older Chinese adults. Thus, tourism could serve as a novel approach to dementia prevention.

## Introduction

Cognitive impairment is characterized by declines in language, attention, and other cognitive functions, most notably memory (1, 2). Cognitive impairment and dementia represent major public health challenges: over 55 million people are living with dementia worldwide, with this figure expected to rise to 78 million by 2030 (3). The incidence of dementia in China was 788.3 per 100,000 people in 2019, exceeding the global rate (682.5 per 100,000) (4). Approximately one-quarter of people with dementia live in China (5). The country’s older adult population is growing as people live longer: China was home to 254 million people above age 60 and 176 million people above age 65 in 2019, representing 17.2 and 12.6% of the general population, respectively (6, 7). Cognitive impairment and dementia, which are common among older adults, thus continue to place increasingly heavy burdens on society.

Cognitive impairment can accompany Alzheimer’s disease, neurological injuries, psychiatric conditions, Parkinson’s disease, and genetic factors, among other causes (8, 9). Given their etiological complexity, no cure currently exists for cognitive impairment and dementia. Approved medications have limited efficacy and substantial side effects (10, 11). Psychosocial and other non-pharmacological therapies (e.g., physical exercise, music therapy, dance movement therapy, nostalgia, and game-based interventions) (12) are preferred to alleviate symptoms (13–16). Of these, recreational therapy has been widely recognized as a useful intervention for people with cognitive decline and dementia (17–19): leisure activities may reduce the risk of cognitive decline and dementia in later life in addition to relieving behavioral and psychological symptoms of dementia (20–23). Tourism, one of the most popular leisure and vacation activities of the 21st century, could offer a way to minimize or even prevent the negative effects of cognitive impairment and dementia on individuals’ health and quality of life (24). However, empirical research on this therapeutic potential is scarce.

Tourism encompasses “the activities of persons traveling to and staying in places outside their usual environment for not more than one consecutive year for leisure, business, and other purposes not related to the exercise of an activity remunerated from within the place visited” (25). Although this domain appears far removed from medical science, the two disciplines are in fact related. Travelers’ inherent mobility facilitates the transmission of infectious diseases and exacerbates public health crises. For instance, the COVID-19 pandemic dealt a devastating blow to the tourism and hospitality industry. Tourism can also play a role in responding to public health priorities (e.g., non-communicable disease epidemics and healthy aging) by enhancing people’s well-being and quality of life (26, 27). As a part of healthy lifestyles, tourism can improve individuals’ physical and mental health through physical activity, social engagement, and positive emotions, potentially contributing to disease prevention and treatment. Tourism is a positive experience, presenting the experiences of relaxing, social interaction, increasing physical activity, seeking knowledge, promoting learning, experiencing different cultures, and escaping routines (28, 29), these activities could energize brain, boost mental health and maintain positive emotion, which are beneficial for cognitive function (30).

Positive tourism experiences may serve as a promising non-pharmacological therapy for multiple populations, such as healthy people, those in suboptimal health, and vulnerable populations with chronic conditions (31, 32). Amid growth in vulnerable groups and their experienced inequities (e.g., stigma, discrimination, exclusion), there is a pressing need for high-quality interdisciplinary research that integrates tourism and medical science. The neglected public health value of tourism can provide a unique perspective on managing cognitive impairment and dementia (33).

Recognition of the benefit of tourism on well-being elevates the demand of older adults for tourism when they tend to have more free time (34). Compared with the younger people, the old people are more actively involved in travel (35). Studies have suggested that active participation in tourism promotes the health condition and longevity of the older adult (36). In details, old tourists have better self-perceived physical and mental health, and better capacity to carry out instrumental activities in daily life than those without tourism experiences (37).

However, there were no studies evidencing the effects of tourism on dementia or cognitive impairment in older adults. Hereby, this study aimed to examine the associations between tourism and cognitive impairment and dementia in older Chinese adults.

## Methods

### Study participants and follow-up

**Figure 1:**
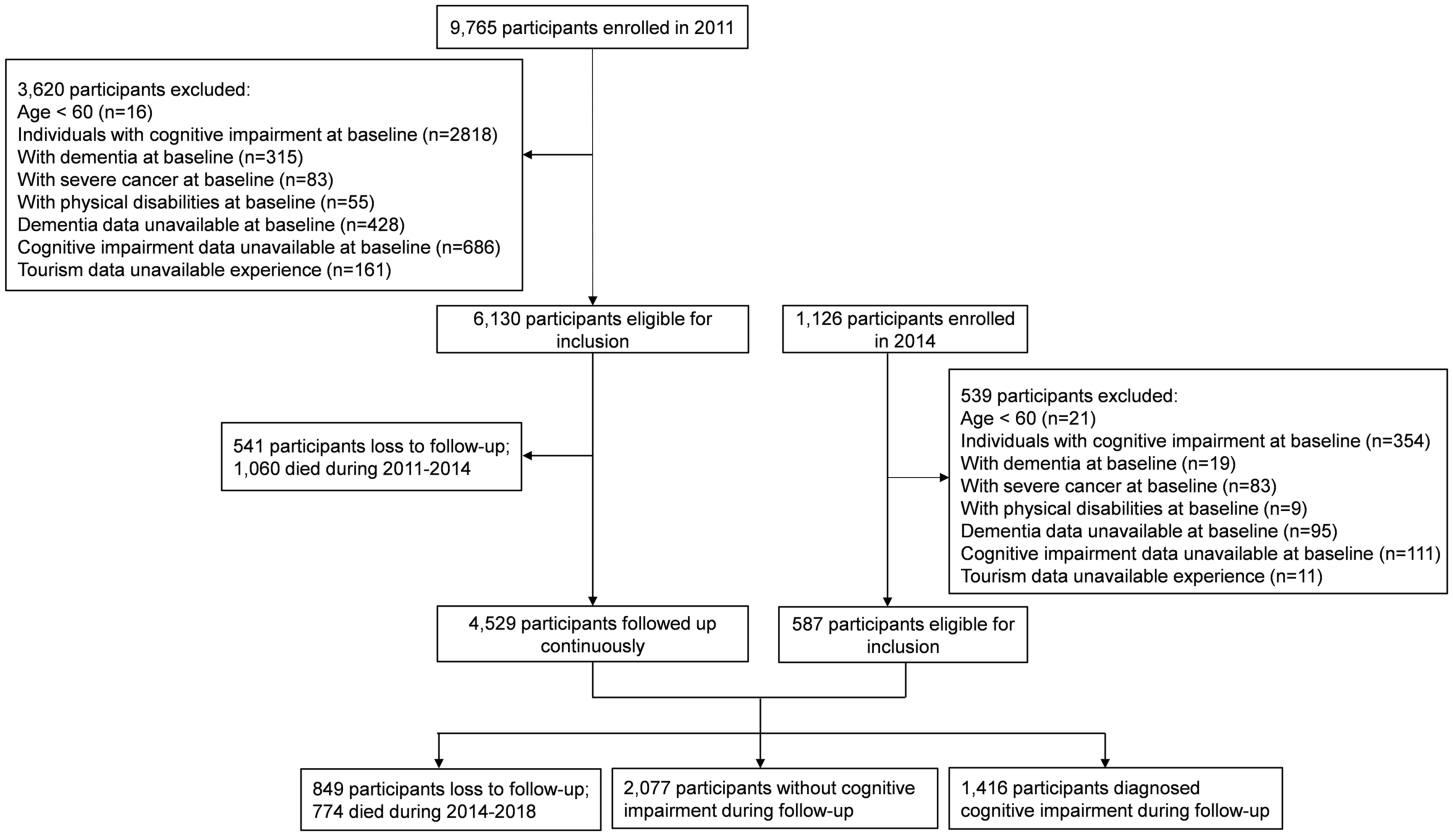
Flowchart of the inclusion of participants.

The Chinese Longitudinal Healthy Longevity Survey (CLHLS) is a nationwide longitudinal cohort study that included older adults from 631 cities across 22 provinces in China between 1998 and 2018 (38). Our study initially enrolled 9,765 participants recruited in 2011 and 1,126 recruited in 2014, who were prospectively followed until 2018. The survey was administered as a face-to-face interview in each participant’s home with assistance from family members, neighbors, or nursing home staff. Several inclusion criteria applied: (1) tourism data available at baseline, (2) no severe somatic or psychological diseases, (3) aged 60 years and above, and (4) normal cognitive function. The following persons were excluded: individuals with (1) cognitive impairment or dementia at baseline, (2) physical disabilities, or (3) other severe somatic or psychological diseases. Data from 6,717 participants were ultimately retained for analysis. All participants were followed from enrollment until 2018. Within this sample, 1,060 participants died and 541 were lost to follow-up from enrollment to the year 2014 when face-to-face interviews were held. During the second stage of follow-up (i.e., 2014–2018), 774 deaths and 849 losses to follow-up were identified before the final visit in 2018. A flow chart depicting the participant selection process appears in Figure 1.

### Measurements

#### Tourism experiences

Information on tourism experiences was obtained at baseline and follow-up using the question “How many times have you traveled outside your home county/city within the past 2 years?.” To investigate the effects of tourism experiences on the incidence of cognitive impairment and dementia, participants were divided into two groups: (1) a tourism group (individuals with at least one tourism experience) and (2) a control group (individuals without tourism experience) (39). In the dose–response analysis, participants with tourism experiences were further classified into three groups: (1) one tourism experience, (2) two tourism experiences, and (3) three or more tourism experiences.

#### Cognitive impairment and dementia

The primary outcome of interest in this cohort study was the onset of cognitive impairment. Cognitive function was measured with the Chinese version of the Mini-Mental State Examination (CMMSE), a modified version of the original MMSE. Considering participants’ low literacy rates, the CMMSE was adapted for comprehensibility (38, 40–42). CMMSE score ≥ 25 was considered as normal cognitive function; those with the score ≤ 24 were with cognitive impairment (41). The severity of cognitive impairment was graded as either mild (18 ≤ CMMSE ≤ 24), moderate (10 ≤ CMMSE ≤ 17), or severe (0 ≤ CMMSE ≤ 9) (43, 44). The secondary outcome of interest was dementia onset. Related information was acquired by referring to participants’ clinical diagnostic reports or disease history.

#### Demographic and socioeconomic characteristics.

Participants’ baseline data included the following: (1) demographics: age, gender, body mass index (BMI), and education level; (2) family/social support: marital status, residence, and living pattern; (3) socioeconomic status: economic status, annual household income; (4) lifestyle and habits: smoking, alcohol consumption, regular physical exercise, sleep quality, and hours of sleep per day.

### Statistical analysis

Normally distributed continuous variables were reported as means and standard deviations, and between-group differences were identified using a student’s *t* test or one-way analysis of variance. Non-normally distributed parameters were listed as a median with interquartile range [P_25_–P_75_] and compared using non-parametric tests. Categorical variables were assessed via chi-square tests. The incidence rates of cognitive impairment and dementia were estimated using the Kaplan–Meier methodology. Moreover, the log-rank test was conducted to identify the difference of survival time between the groups with and without tourism experiences. The Cox proportional hazards model was employed to evaluate the associations between tourism and the incidence of cognitive impairment and dementia, through which hazard ratios (HRs) and their 95% confidence intervals (CIs) were computed. A sensitivity analysis was performed to test the robustness of our estimation by fitting the results to three models: a univariate model (Model 1); a model adjusted for demographics (i.e., age, gender, education level, residence, marital status, and living pattern; Model 2); and a model with further demographic adjustments (i.e., smoking, alcohol consumption, regular physical exercise, BMI, economic status, annual household income, sleep quality, and sleep duration; Model 3) (7). Subgroup analyses were carried out according to participants’ age, gender, marital status, education level, residence, living pattern, smoking, alcohol consumption, regular physical exercise, BMI, sleep quality, sleep duration, economic status, and annual household income. We also explored each interaction between the above factors and tourism experiences in Model 3. All statistical analyses were completed in R Studio (version 4.1.0, R Project for Statistical Computing) and SPSS 26.0 (IBM Corporation, NY, United States). A two-sided *p* value < 0.05 was considered statistically significant.

## Results

### Participants’ baseline characteristics

A total of 6,717 participants (mean age: 81.77 years; 3,125 females, 3,592 males) were included in this study, of whom 669 (9.96%) had had at least one tourism experience in the 2 years prior to enrollment. Participants’ baseline characteristics are listed in Table 1. Nearly half (46.79%) of participants with tourism experiences were between the ages of 70 and 79. Most were married (56.25%), educated (71.75%), lived in urban areas (62.48%), and had a strong economic status (94.47%) and relatively healthy lifestyles.

**Table 1:** Characteristics of the study participants at baseline.

Variables		*N*	Without tourism experiences (%)	With tourism experiences (%)	*p*
Total		6,717	6,048	669	
**Demographics**					
Age (years)	60–69	749	633 (10.47)	116 (17.34)	<0.001
	70–79	2,307	1,994 (33.00)	313 (46.79)	
	80–89	2,020	1,861 (30.77)	159 (23.77)	
	90–99	1,222	1,158 (19.15)	64 (9.57)	
	≥100	419	402 (6.94)	17 (2.54)	
Gender	Male	3,592	3,218 (53.21)	374 (55.90)	0.185
	Female	3,125	2,830 (46.79)	295 (44.10)	
Body mass index (kg/m^2^)	Underweight (<18.5)	1,315	1,223 (22.39)	92 (14.65)	<0.001
	Normal (18.5–24.9)	3,787	3,393 (62.12)	394 (62.74)	
	Overweight (25–29.9)	800	693 (12.69)	107 (17.04)	
	Obese (≥30)	183	153 (2.80)	30 (4.78)	
Education level	Educated	3,502	3,022 (50.36)	480 (71.75)	<0.001
	Uneducated	3,168	2,979 (49.64)	189 (28.25)	
**Family/social support**					
Marital status	Unmarried	72	68 (1.14)	4 (0.71)	0.002
	Married	3,197	2,882 (48.40)	315 (56.25)	
	Divorced or widowed	3,245	3,004 (50.45)	241 (43.04)	
Residence	Living in urban	3,156	2,738 (45.27)	418 (62.48)	<0.001
	Living in rural	3,561	3,310 (54.73)	251 (37.52)	
Living patterns	Living with family members	5,283	4,732 (79.62)	551 (83.36)	0.068
	Living alone/in an institution	1,327	1,211 (20.38)	116 (16.64)	
**Socioeconomic status**					
Economic status	Very rich	105	92 (1.55)	13 (1.94)	<0.001
	Rich	1,142	952 (16.00)	190 (28.40)	
	General	4,567	4,138 (69.53)	429 (64.13)	
	Poor	680	644 (11.16)	36 (5.38)	
	Very poor	126	125 (2.10)	1 (0.15)	
Income (CNY)	<30,000	3,940	3,654 (63.93)	286 (45.83)	<0.001
	30,000~	1,810	1,560 (27.29)	250 (40.06)	
	>80,000	591	503 (8.80)	88 (14.10)	
**Lifestyle and habits**					
Smoking status	Smoker	2,408	2,146 (35.84)	262 (39.16)	0.090
	Non-smoker	4,249	3,842 (64.16)	407 (60.84)	
Alcohol drinking	Drinker	1,976	1,727 (28.91)	249 (37.22)	<0.001
	Non-drinker	4,696	4,246 (71.09)	450 (62.78)	
Regular exercise	Yes	1,824	1,540 (25.95)	284 (42.84)	<0.001
	No	4,774	4,395 (74.05)	379 (57.16)	
Sleep time (hours)	<6	994	909 (15.03)	85 (12.70)	0.012
	6–10	5,315	4,758 (78.67)	557 (83.26)	
	>10	408	381 (6.30)	27 (4.04)	
Sleep quality	Very good	1,221	1,042 (17.29)	179 (26.76)	<0.001
	Good	3,115	2,832 (47.00)	283 (42.30)	
	General	1,626	1,482 (24.60)	144 (21.52)	
	Bad	669	609 (10.11)	60 (9.00)	
	Very bad	63	60 (1.00)	3 (0.45)	

CNY, Chinese Yuan.

### Association between tourism experiences and cognitive impairment

The median follow-up time was 48.12 months (47.40 months for participants without tourism experiences and 55.20 months for participants with tourism experiences). Of the 669 participants with tourism experiences, 97 (14.50%) developed cognitive impairment compared with 1,319 (21.81%) of the 6,048 participants without such experiences. The incidence density (ID) of cognitive impairment was lower in participants with tourism experiences (ID = 316.94 per 10,000 person-years) than in those without (ID = 552.38 per 10,000 person-years) (Table 2).

**Table 2:** Association between tourism experiences and cognitive impairment.

Tourism experiences	Incidence	Incidence density (per 10,000 person–years)	Model 1		Model 2		Model 3	
			cHR (95% CI)	*p*	aHR (95% CI)	*p*	aHR (95% CI)	*p*
0 tourism experience	1,319/6,048	552.38	1 (reference)		1 (reference)		1 (reference)	
≥1 tourism experiences	97/669	316.94	0.41 (0.41–0.62)	<0.001	0.68 (0.55–0.84)	<0.001	0.69 (0.55–0.87)	0.0020

CI, confidence interval; cHR, crude hazard ratio; aHR, adjusted hazard ratio; Model 1 is a univariate model; Model 2, adjusted for age, gender, education level, residence, marital status, and living pattern; Model 3, adjusted for all covariates in Model 2, and smoking, alcohol drinking, regular exercise, BMI, economic status, annual income, and sleeping status.

**Figure 2:**
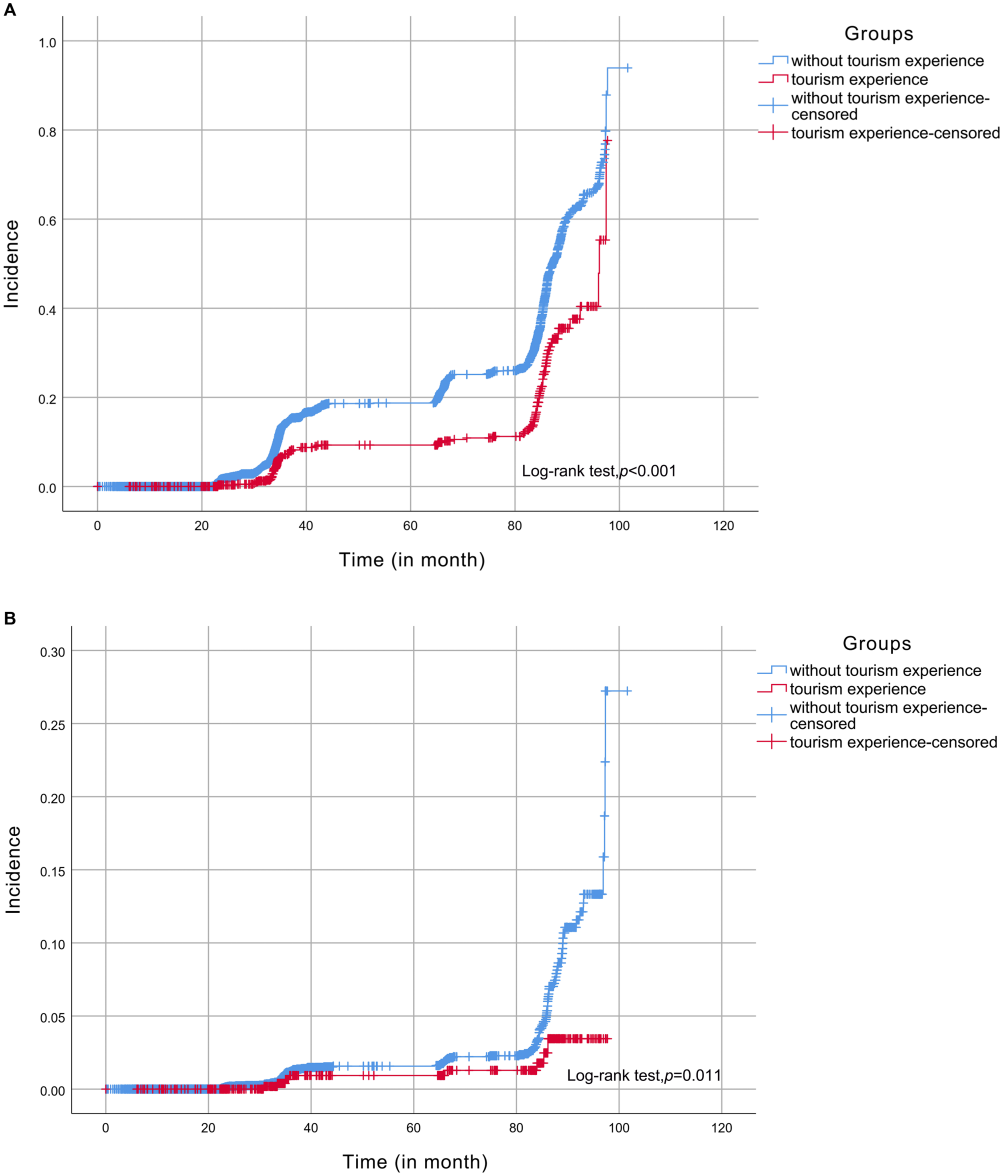
Kaplan-Meier analysis; **(A)** cognitive impairment, **(B)** dementia.

As shown in Figure 2A, the Kaplan–Meier analysis illustrated that individuals with tourism experiences had lower incidence cognitive impairment compared with those without tourism experiences. In the univariate analysis, participants with tourism experiences were at a lower risk of cognitive impairment (crude hazard ratio [cHR] = 0.51, 95% CI: 0.41–0.62, *p* < 0.001). In Model 2, after adjusting for age, gender, education level, residence, marital status, and living pattern, a lower risk of cognitive impairment was also observed (adjusted hazard ratio [aHR] = 0.68, 95%CI: 0.55–0.84, *p* < 0.001) in people with tourism experiences. Upon adjusting for all covariates (i.e., Model 3), we observed a significantly lower risk of cognitive impairment (aHR: 0.69, 95% CI: 0.55–0.87, *p* = 0.002) in this group.

**Figure 3:**
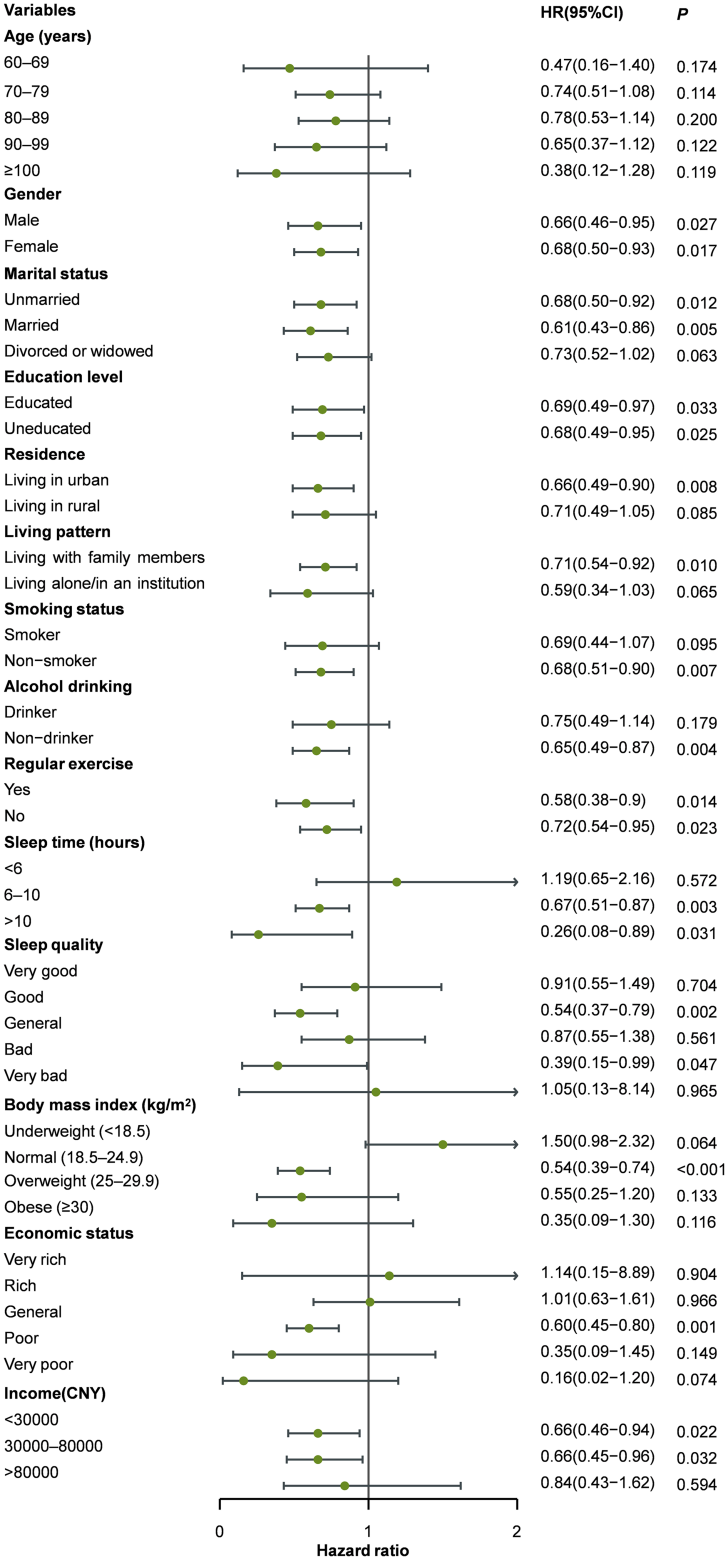
Subgroup analysis on cognitive impairment.

During subgroup analysis, on the bases of age, gender, education level, residence, marital status, living pattern, smoking, alcohol consumption, regular physical exercise, BMI, economic status, annual household income, sleep quality, and hours slept per day, the association between the risk of cognitive impairment and tourism experiences was robust (Supplementary Table S1; Figure 3). The estimates of interaction between tourism experiences and covariates were not significant (Supplementary Table S1).

**Figure 4:**
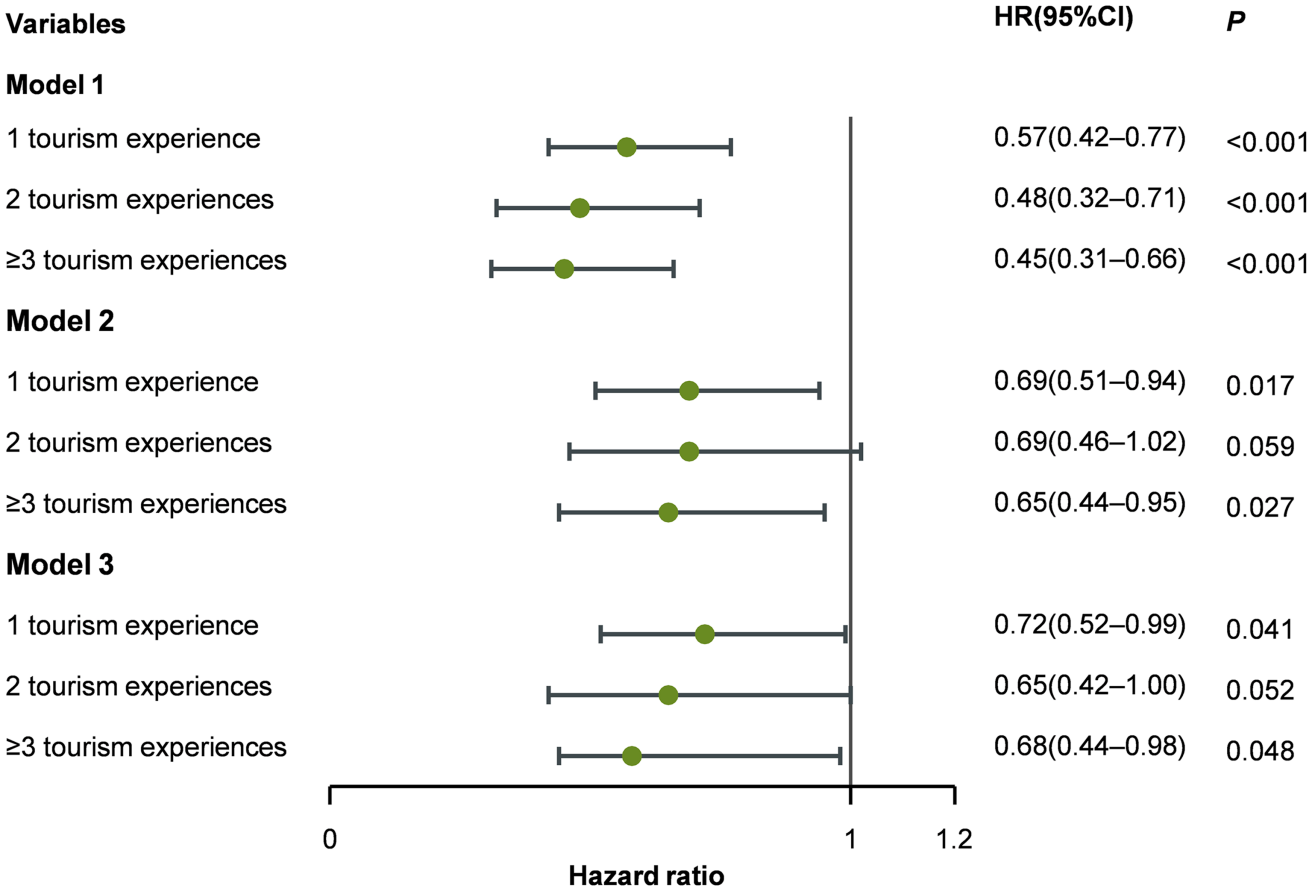
Dose-response association between tourism experiences and cognitive impairment.

Regarding dose–response analysis (Supplementary Table S2; Figure 4), among participants with ≥3 tourism experiences, the HR for cognitive impairment was 0.45 (95% CI: 0.31–0.66)—lower than the HRs for individuals with two tourism experiences (HR = 0.48, 95% CI: 0.32–0.71) or one tourism experience (HR = 0.57, 95% CI: 0.42–0.77). The linear dose–response manner was significant (*Z* = −4.218, *p* < 0.001). Multivariate analysis revealed that tourism frequency influenced the incidence of cognitive impairment in a dose–response manner.

In terms of the severity of cognitive impairment, we compared CMMSE scores between participants with and without tourism experiences. As indicated in Supplementary Table S3, the impairment severity was significantly higher for individuals with tourism experiences than for those without.

### Association between tourism experiences and dementia

Of the 669 participants with tourism experiences, only 8 (1.20%) developed dementia, whereas 131 (2.17%) of the 6,048 participants without such experiences were diagnosed with dementia. The ID of dementia in participants with tourism experiences (ID = 26.14 per 10,000 person-years) was lower than in participants without tourism experiences (ID = 54.86 per 10,000 person-years) (Table 3). The Kaplan–Meier analysis indicated that the incidence of dementia was significantly lower among individuals with tourism experiences than those without tourism experiences (Figure 2B). Univariate analysis demonstrated that participants with tourism experiences had a lower risk of dementia (cHR = 0.41, 95% CI: 0.20–0.84, *p* = 0.014) than those without such experiences. In Model 2, upon adjusting for age, gender, education level, residence, marital status, and living pattern, a lower risk of dementia was also observed (aHR = 0.47, 95% CI: 0.23–0.96, *p* = 0.038). In Model 3, a significantly lower risk of dementia (aHR: 0.41, 95% CI: 0.19–0.89, *p* = 0.024) was found after adjusting for all covariates. Supplementary Table S4; Supplementary Figure S1 illustrate that the dose–response analysis yielded an insignificant association between the risk of dementia and tourism frequency (*Z* = −1.514, *p* = 0.130). In the subgroup analysis, the association between tourism experiences and the risk of dementia was strong (Supplementary Table S5; Supplementary Figure S2). We did not observe significant interactions between tourism experiences and the covariates (Supplementary Table S5).

**Table 3:** Association of tourism experiences with dementia.

Tourism experiences	Incidence	Incidence density (Per 10,000 person–years)	Model 1		Model 2		Model 3	
			cHR (95% CI)	*p*	aHR (95% CI)	*p*	aHR (95% CI)	*p*
0 tourism experience	131/6,048	54.86	1 (reference)		1 (reference)		1 (reference)	
≥1 tourism experiences	8/669	26.14	0.41 (0.20–0.84)	0.014	0.47 (0.23–0.96)	0.038	0.41 (0.19–0.89)	0.024

CI, confidence interval; cHR, crude hazard ratio; aHR, adjusted hazard ratio; Model 1 is a univariate model; Model 2, adjusted for age, gender, education level, residence, marital status, and living pattern; Model 3, adjusted for all covariates in Model 2, and smoking, alcohol drinking, regular exercise, BMI, economic status, annual income and sleeping status.

## Discussion

Our findings showed that tourism experiences reduced the incidence of cognitive impairment and dementia in older Chinese adults. Tourism may even protect against cognitive decline in a dose–response manner. Although tourism has been broadly acknowledged as beneficial to physical and mental health, few studies have examined the protective effects of tourism on cognitive function (24, 45). This nationwide prospective cohort study revealed a correlation between tourism experiences along with declines in cognitive impairment and dementia.

As a multi-component form of leisure, tourism can improve physical and mental health (especially cognitive function) through physical activity, positive emotions, social engagement, therapeutic landscapes (e.g., nature-based tourism, culture-based tourism) and other means. Tourism requires mobility, especially when outdoors, is inextricably linked with physical activity (e.g., walking, hiking, mountain climbing, cycling) (39, 46, 47), therefore supports physical activity (48). Studies have shown that non-participation in tourism is associated with low physical activity (49). Physical activity has numerous positive impacts on physical and mental health, effectively reducing poor health outcomes (50, 51). A systematic review summarized the roles of physical activity in improved fitness and cognitive function and indicated that people with moderate daily physical activity had better physical performance and fewer signs of cognitive delay than people who engaged in less physical activity (52). Evidence further suggests that physical activity affects cognitive function in a dose–response manner, indicating that more intense physical activity correlates with better cognitive function (53–55). A 6-month physical activity intervention was also reported to produce significant but modest enhancements in cognitive function in older adults at high risk of dementia (56). Physical activity is a prominent supportive treatment for cognitive impairment.

Autonomic dysfunction is closely related to the development of dementia (57). Engaging in physical activity and avoiding prolonged sedentary behavior can help maintain the normal function of the autonomic nervous system (ANS) (58). Taking part in domestic nature-based tourism can improve ANS functioning via stress reduction (59). Decreased dopamine levels are associated with dementia and Parkinson’s disease (60), but physical activity can prompt dopamine release and maintain a younger biological age (61, 62). Promoting physical activity through travel, especially outdoors, can therefore contribute to better health and a higher quality of life. Tourism sites that are popular among older travelers should host creative activities to encourage physical movement, particularly for individuals with limited mobility. For example, drum circles and chair dance programs could enable mobility-limited travelers to engage in physical activity while visiting nature centers or other outdoor venues. Older travelers with good mobility could meander along nature trails or take walks on easy terrain to enhance their physical activity while traveling.

Poor psychological functioning also increases dementia risk (63–65). A pleasant mood can upregulate dopamine secretion in tourists, playing an important role in cognitive health (66). Tourists have great access to green environments (e.g., parks, lakes, hills, forests) and friendly people to alleviate stress and maintain a pleasant mental state (67). For instance, forest bathing is a natural therapy to assuage negative emotions and improve bodily functions (68). A large body of evidence indicates that people who regularly participate in tourism activities have better mental well-being than those who do not (69). A cohort study found that people in a poor mood were at higher risk of dementia than those in a better mood (70). Positive tourism experiences relax tourists’ bodies and minds, elicit optimism and pleasant emotions (71), and enhance cognitive function (39). Our study showed that tourism can lessen the symptoms of cognitive impairment and dementia; positive emotions arising during travel may play considerable roles in this improvement. Therefore, tourism and hospitality practitioners should aim to evoke positive emotions among travelers by offering high-quality products and services (e.g., vulnerability-friendly amenities, personalized tours) and meaningful experiences (e.g., cultural immersion, social interaction).

Social engagement is similarly vital for maintaining cognitive function. Greater social engagement reduces dementia risk in the older adult (72, 73). This type of engagement can delay or prevent dementia onset even in people at high risk of cognitive impairment (74). In contrast to physical activity, social engagement allows tourists to interact with their surroundings (e.g., other travelers, residents, tour guides, animals) (75), thus strengthening their connection to society (76). Encouraging older adults to interact socially can ameliorate loneliness and depression, ultimately improving mental health and cognitive function (77). Tourism and hospitality practitioners should take various measures to promote social engagement in destinations: social spaces (e.g., public squares, gardens) and interactive activities (e.g., festivals and events, volunteer activities, interactive technologies) should be coordinated. Front desk attendants at venues and hotels can be instructed to facilitate social engagement at destinations that are welcoming of older travelers.

### Limitations

This cohort study demonstrated the preventive effect of tourism on cognitive impairment among the older adult; however, there are several limitations. First, findings from a Chinese population might not generalize elsewhere due to socioeconomic circumstances and lifestyles. Second, we did not investigate tourism activities in depth (e.g., length of tourism experience, and types of tourism). The specific effects of different components of tourism on cognitive impairment and dementia also remain to be further explored. Third, although multivariate analyses were conducted to adjust for regular physical exercise and other covariates, participants’ baseline health status could have biased our findings: we excluded individuals with Parkinson’s disease, physical disabilities, and other severe somatic diseases. In addition, the participants were most married, educated, lived in urban areas and in a strong economic status, which contributed to mitigation of the progress of cognitive impairment. We cannot fully control for these confounding factors that influence the contribution of tourism experience.

## Conclusion

Our nationwide prospective cohort analysis documented correlations between tourism experiences and a lower risk of cognitive impairment and dementia. Results suggest that tourism can enhance cognitive function and should be considered as a non-pharmacological intervention to prevent cognitive impairment and dementia.

## Data availability statement

The original contributions presented in the study are included in the article/Supplementary material, further inquiries can be directed to the corresponding author.

## Ethics statement

The studies involving humans were approved by Ethical Review Committee of Peking University (IRB00001052–13074). The studies were conducted in accordance with the local legislation and institutional requirements. Written informed consent for participation was not required from the participants or the participants’ legal guardians/next of kin in accordance with the national legislation and institutional requirements.

## Author contributions

QL: Software, Writing – original draft, Data curation. ZG: Writing – original draft, Software. FH: Writing – original draft, Data curation. MX: Writing – original draft, Data curation, Software. QZ: Writing – review & editing, Conceptualization. JW: Writing – review & editing, Conceptualization, Methodology. TY: Writing – review & editing. DZ: Writing – review & editing, Formal analysis. YW: Resources, Writing – review & editing, Formal analysis. SY: Writing – review & editing, Formal analysis. HH: Writing – review & editing, Conceptualization, Formal analysis.
