# Supplementary figures and images for: Tourism experiences reduce the risk of cognitive impairment in the Chinese older adult: a prospective cohort study
Source: Front Public Health. 2023 Oct 24;11:1271319. doi: 10.3389/fpubh.2023.1271319 (PMC10629014; doi:10.3389/fpubh.2023.1271319)

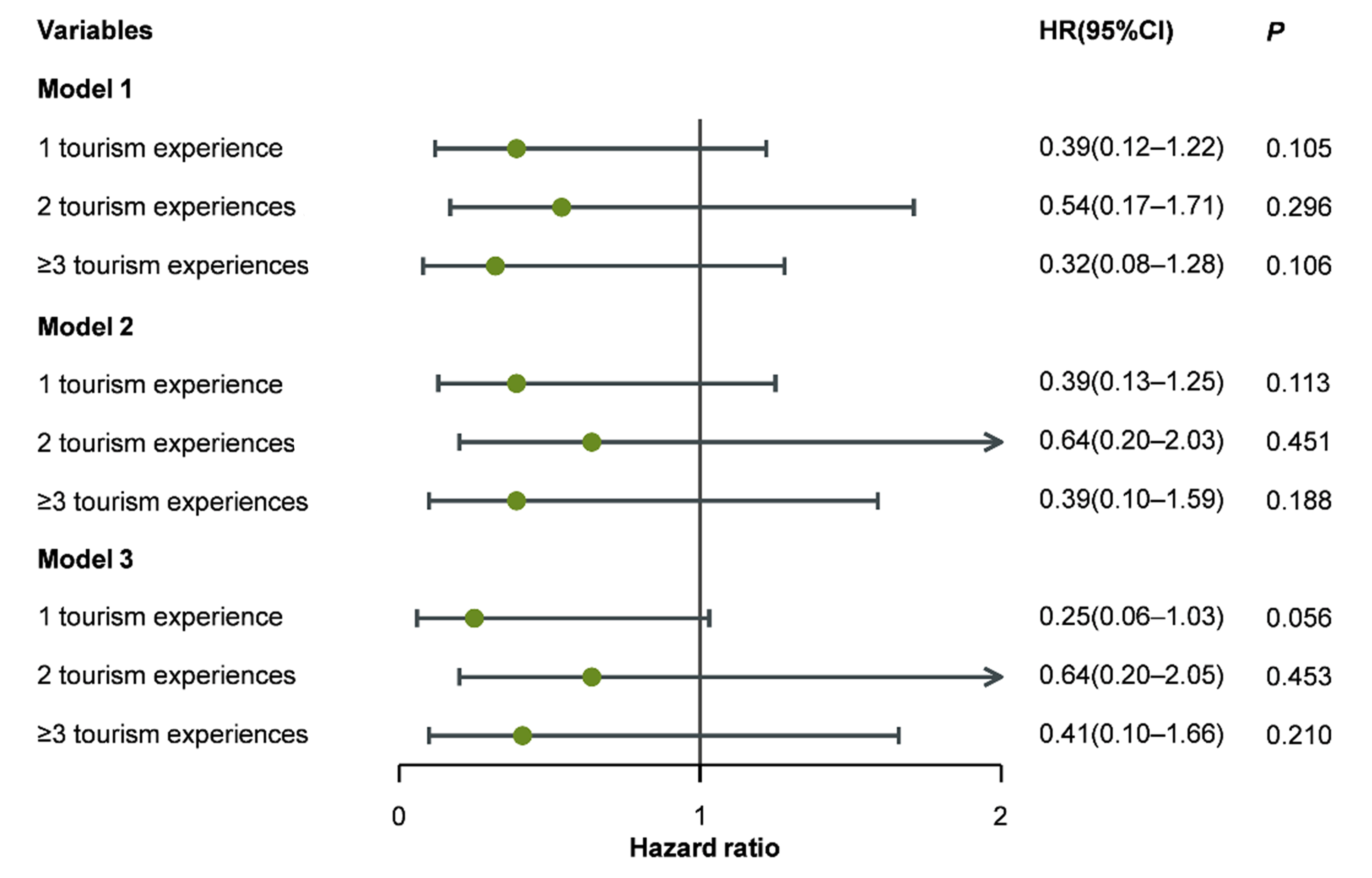

Supplement: Supplementary file 1 [file Image_1.TIF]

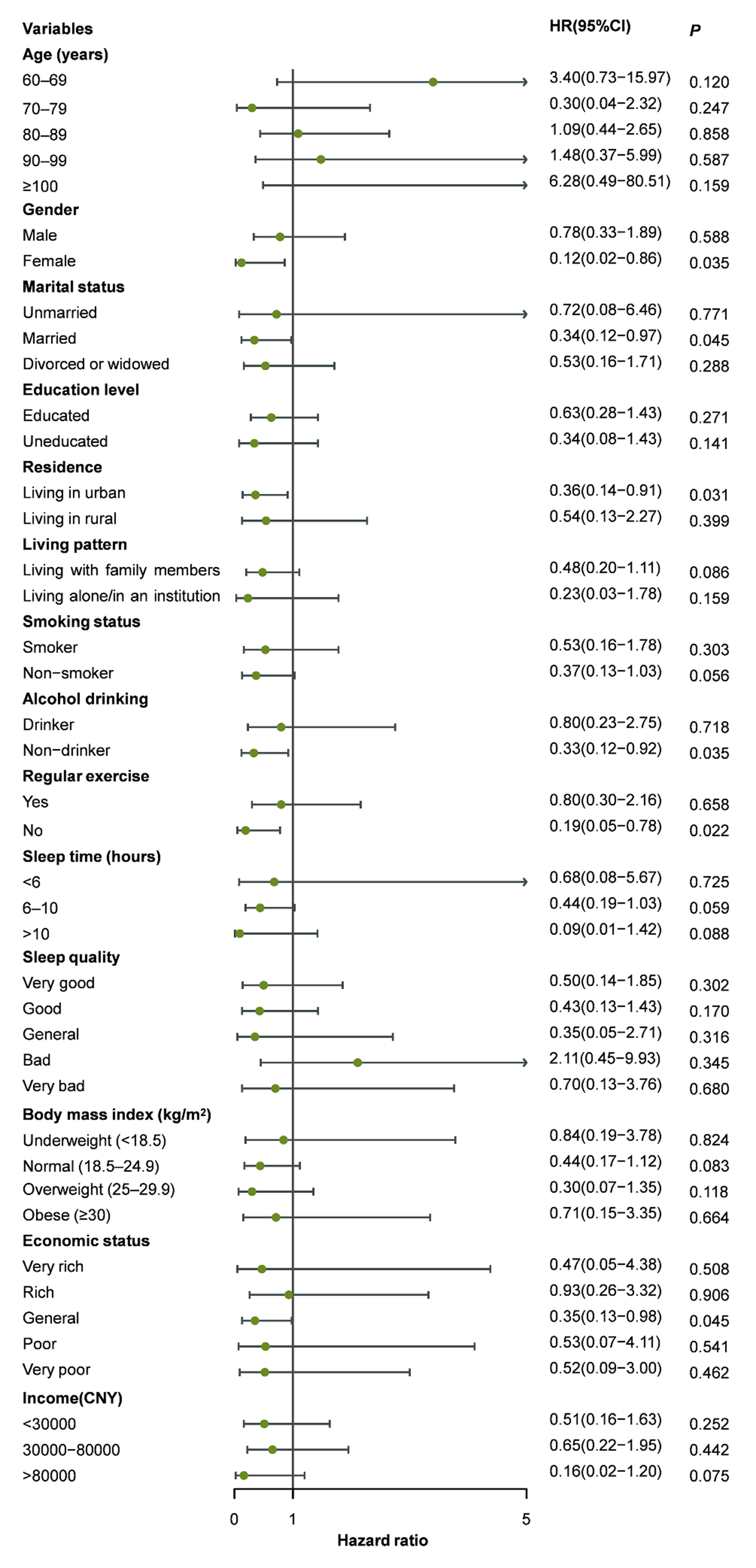

Supplement: Supplementary file 2 [file Image_2.TIF]
