# Supplementary material for: Tourism experiences reduce the risk of cognitive impairment in the Chinese older adult: a prospective cohort study
Source: Front Public Health. 2023 Oct 24;11:1271319. doi: 10.3389/fpubh.2023.1271319 (PMC10629014; doi:10.3389/fpubh.2023.1271319)
Supplement: Supplementary file 3 [file Table_1.DOCX]

**[Supplementary materials]**

**Table S1** **Subgroup analysis on cognitive impairment**

| **Variables** |  | **aHR (95%CI)** | ***P*** | ***P*-interaction** |
| --- | --- | --- | --- | --- |
| **Demographics** |  |  |  |  |
| Age (years) | 60–69 | 0.47(0.16–1.40) | 0.174 | 0.858 |
|  | 70–79 | 0.74(0.51–1.08) | 0.114 |  |
|  | 80–89 | 0.78(0.53–1.14) | 0.200 |  |
|  | 90–99 | 0.65(0.37–1.12) | 0.122 |  |
|  | ≥100 | 0.38(0.12–1.28) | 0.119 |  |
| Gender | Male | 0.66(0.46–0.95) | 0.027 | 0.171 |
|  | Female | 0.68(0.50–0.93) | 0.017 |  |
| Body mass index (kg/m^2^) | Underweight (<18.5) | 1.50(0.98–2.32) | 0.064 | 0.263 |
|  | Normal (18.5–24.9) | 0.54(0.39–0.74) | <0.001 |  |
|  | Overweight (25–29.9) | 0.55(0.25–1.20) | 0.133 |  |
|  | Obese (≥30) | 0.35(0.09–1.30) | 0.116 |  |
| Education level | Educated | 0.69(0.49–0.97) | 0.033 | 0.400 |
|  | Uneducated | 0.68(0.49–0.95) | 0.025 |  |
| **Family/social support** |  |  |  |  |
| Marital status, n (%) | Unmarried | 0.68(0.5–0.92) | 0.012 | 0.522 |
|  | Married | 0.61(0.43–0.86) | 0.005 |  |
|  | Divorced or widowed | 0.73(0.52–1.02) | 0.063 |  |
| Residence | Living in urban | 0.66(0.49–0.90) | 0.008 | 0.667 |
|  | Living in rural | 0.71(0.49–1.05) | 0.085 |  |
| Living patterns | Living with family members | 0.71(0.54–0.92) | 0.010 | 0.263 |
|  | Living alone/in an institution | 0.59(0.34–1.03) | 0.065 |  |
| **Socioeconomic status** |  |  |  |  |
| Economic status | Very rich | 1.14(0.15–8.89) | 0.904 | 0.781 |
|  | Rich | 1.01(0.63–1.61) | 0.966 |  |
|  | General | 0.60(0.45–0.80) | 0.001 |  |
|  | Poor | 0.35(0.09–1.45) | 0.149 |  |
|  | Very poor | 0.16(0.02–1.20) | 0.074 |  |
| Income (CNY) | <30000 | 0.66(0.46–0.94) | 0.022 | 0.863 |
|  | 30000–80000 | 0.66(0.45–0.96) | 0.032 |  |
|  | >80000 | 0.84(0.43–1.62) | 0.594 |  |
| **lifestyle and habits** |  |  |  |  |
| Smoking status | Smoker | 0.69(0.44–1.07) | 0.095 | 0.662 |
|  | Non-smoker | 0.68(0.51–0.90) | 0.007 |  |
| Alcohol drinking | Drinker | 0.75(0.49–1.14) | 0.179 | 0.306 |
|  | Non-drinker | 0.65(0.49–0.87) | 0.004 |  |
| Regular exercise | Yes | 0.58(0.38–0.90) | 0.014 | 0.537 |
|  | No | 0.72(0.54–0.95) | 0.023 |  |
| Sleep time (hours) | <6 | 1.19(0.65–2.16) | 0.572 | 0.071 |
|  | 6–10 | 0.67(0.51–0.87) | 0.003 |  |
|  | >10 | 0.26(0.08–0.89) | 0.031 |  |
| Sleep quality | Very good | 0.91(0.55–1.49) | 0.704 | 0.973 |
|  | Good | 0.54(0.37–0.79) | 0.002 |  |
|  | General | 0.87(0.55–1.38) | 0.561 |  |
|  | Bad | 0.39(0.15–0.99) | 0.047 |  |
|  | Very bad | 1.05(0.13–8.14) | 0.965 |  |

CI: confidence interval, aHR, hazard ratio adjusted for age, gender, education level, residence, marital status, and living pattern, smoking, alcohol drinking, regular exercise, BMI, economic status, annual income and sleeping status; *P*-interaction, P-value for interaction between tourism and each variable.

**Table S2 Dose–response association between tourism experiences and cognitive impairment**

| **Tourism experiences** | **Incidence** | **Incidence density,**  **per 10,000 person–years** | **Model 1** |  |  | **Model 2** |  |  | **Model 3** |  |
| --- | --- | --- | --- | --- | --- | --- | --- | --- | --- | --- |
|  |  |  | cHR (95% CI) | *P* |  | aHR (95% CI) | *P* |  | aHR (95% CI) | *P* |
| 0 tourism experience | 1319/6048 | 552.38 | 1 (reference) |  |  | 1 (reference) |  |  | 1 (reference) |  |
| 1 tourism experience | 44/282 | 349.35 | 0.57  (0.42–0.77) | <0.001 |  | 0.69  (0.51–0.94) | 0.017 |  | 0.72  (0.52–0.99) | 0.041 |
| 2 tourism experiences | 26/188 | 295.82 | 0.48  (0.32–0.71) | <0.001 |  | 0.69  (0.46–1.02) | 0.059 |  | 0.65  (0.42–1.01) | 0.052 |
| ≥3 tourism experiences | 27/199 | 292.69 | 0.45  (0.31–0.66) | <0.001 |  | 0.65  (0.44–0.95) | 0.027 |  | 0.68  (0.44–0.98) | 0.048 |

CI, confidence interval; cHR, crude hazard ratio; aHR, adjusted hazard ratio; Model 1 is a univariate model; Model 2, adjusted for basic characteristics，including age, gender, education level, residence, marital status, and living pattern; Model 3, adjusted for all covariates in Model 2, and smoking, alcohol drinking, regular exercise, BMI, economic status, annual income and sleeping status.

**Table S****3 Association of tourism experiences with the severity of cognitive impairment in the multivariable model**

| **Tourism experiences** | **Mild cognitive impairment** | | |  | **Moderate cognitive impairment** | | |  | **Severe cognitive impairment** | | |
| --- | --- | --- | --- | --- | --- | --- | --- | --- | --- | --- | --- |
|  | Incidence | aHR (95% CI) | *P* |  | Incidence | aHR(95% CI) | *P* |  | Incidence | aHR(95% CI) | *P* |
| 0 tourism experience | 724/5453 | 1 (reference) | **–** |  | 298/5027 | 1 (reference) | **–** |  | 321/5050 | 1 (reference) | **–** |
| ≥ 1 tourism experiences | 64/636 | 0.71  (0.53–0.94) | 0.019 |  | 15/587 | 0.44  (0.25–0.80) | 0.0070 |  | 20/592 | 0.58  (0.36–0.94) | 0.026 |

CI, confidence interval; aHR, adjusted hazard ratio, adjusted for age, gender, education level, residence, marital status, and living pattern, smoking, alcohol drinking, regular exercise, BMI, economic status, annual income and sleeping status.

**Table S4** **Dose–response association between tourism experiences and dementia**

| **Tourism experiences** | **Incidence** | **Incidence density,**  **per 10,000 person–years** | **Model 1** |  |  | **Model 2** |  |  | **Model 3** |  |
| --- | --- | --- | --- | --- | --- | --- | --- | --- | --- | --- |
|  |  |  | cHR (95% CI) | *P* |  | aHR (95% CI) | *P* |  | aHR (95% CI) | *P* |
| 0 tourism experience | 131/6048 | 54.86 | 1 (reference) |  |  | 1 (reference) |  |  | 1 (reference) |  |
| 1 tourism experience | 3/282 | 23.83 | 0.39  (0.12–1.22) | 0.105 |  | 0.39  (0.13–1.25) | 0.113 |  | 0.25  (0.06–1.03) | 0.056 |
| 2 tourism experiences | 3/188 | 34.18 | 0.54  (0.17–1.71) | 0.296 |  | 0.64  (0.20–2.03) | 0.451 |  | 0.64  (0.20–2.05) | 0.453 |
| ≥3 tourism experiences | 2/199 | 21.26 | 0.31  (0.08–1.28) | 0.106 |  | 0.39  (0.10–1.59) | 0.188 |  | 0.41  (0.10–1.66) | 0.210 |

CI, confidence interval; cHR, crude hazard ratio; aHR, adjusted hazard ratio; Model 1, a univariate model; Model 2, adjusted for basic characteristics，including age, gender, education level, residence, marital status, and living pattern; Model 3, adjusted for all covariates in Model 2, and smoking, alcohol drinking, regular exercise, BMI, economic status, annual income and sleeping status.

**Table S5** **Subgroup analysis on dementia**

| **Variables** |  | **aHR (95%CI)** | ***P*** | ***P*-interaction** |
| --- | --- | --- | --- | --- |
| **Demographics** |  |  |  |  |
| Age (years) | 60–69 | 3.40(0.73–15.97) | 0.120 | 0.145 |
|  | 70–79 | 0.30(0.04–2.32) | 0.247 |  |
|  | 80–89 | 1.09(0.44–2.65) | 0.858 |  |
|  | 90–99 | 1.48(0.37–5.99) | 0.587 |  |
|  | ≥100 | 6.28(0.49–80.51) | 0.159 |  |
| Gender | Male | 0.78(0.33–1.89) | 0.588 | 0.054 |
|  | Female | 0.12(0.02–0.86) | 0.035 |  |
| Body mass index (kg/m^2^) | Underweight (<18.5) | 0.84(0.19–3.78) | 0.824 | 0.138 |
|  | Normal (18.5–24.9) | 0.44(0.17–1.12) | 0.083 |  |
|  | Overweight (25–29.9) | 0.3(0.07–1.35) | 0.118 |  |
|  | Obese (≥30) | 0.71(0.15–3.35) | 0.664 |  |
| Education level | Educated | 0.63(0.28–1.43) | 0.271 | 0.293 |
|  | Uneducated | 0.34(0.08–1.43) | 0.141 |  |
| **Family/social support** |  |  |  |  |
| Marital status, n (%) | Unmarried | 0.72(0.08–6.46) | 0.771 | 0.866 |
|  | Married | 0.34(0.12–0.97) | 0.045 |  |
|  | Divorced or widowed | 0.53(0.16–1.71) | 0.288 |  |
| Residence | Living in urban | 0.36(0.14–0.91) | 0.031 | 0.782 |
|  | Living in rural | 0.54(0.13–2.27) | 0.399 |  |
| Living patterns | Living with family members | 0.48(0.2–1.11) | 0.086 | 0.442 |
|  | Living alone/in an institution | 0.23(0.03–1.78) | 0.159 |  |
| **Socioeconomic status** |  |  |  |  |
| Economic status | Very rich | 0.47(0.05–4.38) | 0.508 | 0.358 |
|  | Rich | 0.93(0.26–3.32) | 0.906 |  |
|  | General | 0.35(0.13–0.98) | 0.045 |  |
|  | Poor | 0.53(0.07–4.11) | 0.541 |  |
|  | Very poor | 0.52(0.09–3) | 0.462 |  |
| Income (CNY) | <30000 | 0.51(0.16–1.63) | 0.252 | 0.599 |
|  | 30000–80000 | 0.65(0.22–1.95) | 0.442 |  |
|  | ＞80000 | 0.16(0.02–1.2) | 0.075 |  |
| **lifestyle and habits** |  |  |  |  |
| Smoking status | Smoker | 0.53(0.16–1.78) | 0.303 | 0.989 |
|  | Non-smoker | 0.37(0.13–1.03) | 0.056 |  |
| Alcohol drinking | Drinker | 0.8(0.23–2.75) | 0.718 | 0.790 |
|  | Non-drinker | 0.33(0.12–0.92) | 0.035 |  |
| Regular exercise | Yes | 0.8(0.3–2.16) | 0.658 | 0.115 |
|  | No | 0.19(0.05–0.78) | 0.022 |  |
| Sleep time (hours) | <6 | 0.68(0.08–5.67) | 0.725 | 0.593 |
|  | 6–10 | 0.44(0.19–1.03) | 0.059 |  |
|  | >10 | 0.09(0.01–1.42) | 0.088 |  |
| Sleep quality | Very good | 0.5(0.14–1.85) | 0.302 | 0.104 |
|  | Good | 0.43(0.13–1.43) | 0.17 |  |
|  | General | 0.35(0.05–2.71) | 0.316 |  |
|  | Bad | 2.11(0.45–9.93) | 0.345 |  |
|  | Very bad | 0.7(0.13–3.76) | 0.68 |  |

HR: hazard ratio; CI: confidence interval; CNY, Chinese Yuan; aHR, adjusted for age, gender, education level, residence, marital status, and living pattern, smoking, alcohol drinking, regular exercise, BMI, economic status, annual income and sleeping status; *P*-interaction, the *P*-value for interaction between tourism and each variable.
